# Cardiorenal Syndrome in Adults with Congenital Heart Disease

**DOI:** 10.3390/jcm14134392

**Published:** 2025-06-20

**Authors:** Shailendra Upadhyay, Anudeep K. Dodeja, Olga Toro-Salazar, Whitney Fairchild, Frank Han

**Affiliations:** 1 Cardiology Department, Connecticut Children’s, Hartford, CT 06106, USA; adodeja@connecticutchildrens.org (A.K.D.); otoro@connecticutchildrens.org (O.T.-S.); wfairchild@connecticutchildrens.org (W.F.); fhan@connecticutchildrens.org (F.H.); 2 Department of Pediatrics and Pediatric Cardiology, University of Connecticut School of Medicine, Farmington, CT 06030, USA

**Keywords:** adult congenital heart disease, cardio renal syndrome, systemic right ventricle, Fontan circulation, heart failure

## Abstract

As the population of adults with congenital heart disease (ACHD) continues to grow, a significant and often underrecognized complication is the development of cardiorenal syndrome (CRS)—a complex, bidirectional interaction between cardiac and renal dysfunction. While CRS has been extensively studied in acquired heart failure, its manifestations and implications in ACHD remain insufficiently understood. Emerging data suggest that renal dysfunction is highly prevalent in ACHD, with significant associations to adverse outcomes regardless of cardiac lesion type or functional status. This review explores CRS within three key physiologic categories in ACHD: patients with a systemic right ventricle, those with a subpulmonary right ventricle, and those with Fontan circulation. Each subgroup presents unique hemodynamic challenges that affect renal perfusion, filtration pressure, and systemic congestion, contributing to both acute and chronic renal impairment. The utility of renal biomarkers such as albuminuria, cystatin C, and estimated glomerular filtration rate (eGFR) is emphasized, alongside the importance of early detection and multidisciplinary management. Heart failure therapy tailored to congenital anatomy, neurohormonal modulation, and careful volume control remain the cornerstones of treatment, while transplantation strategies must consider the potential for irreversible end-organ damage. Given the profound implications of CRS on quality of life and survival, a comprehensive understanding of its pathophysiology and management in ACHD is critical to optimizing long-term outcomes in this increasingly complex patient population.

## 1. Introduction

Advances in congenital heart disease (CHD) care have produced a rapidly expanding population of adult survivors. As these patients age, heart failure (HF) has emerged as a leading cause of morbidity and mortality in ACHD, with roughly 17–42% of late deaths attributed to HF in some series [1]. In parallel, chronic kidney disease (CKD) is increasingly recognized in ACHD patients as an important comorbidity. Renal dysfunction often develops insidiously, but even mild impairment in renal function has been shown to adversely impact cardiovascular outcomes [2]. The concept of cardiorenal syndrome (CRS) encompasses the complex, bidirectional interactions whereby chronic cardiac dysfunction can lead to kidney dysfunction and vice versa. In acquired HF populations, CRS confers worse survival and complicates management. Emerging evidence indicates ACHD patients are similarly—if not more—vulnerable to CRS due to lifelong hemodynamic stresses, surgical scars, hypoxemia, and neurohormonal activation.

Epidemiologic data underscore the high prevalence of renal impairment in ACHD. Contemporary studies estimate that approximately 30–50% of adult CHD patients have significantly reduced renal function [3]. In one large cohort of 1102 ACHD patients (median age 36 years), 50% had a GFR below normal (<90 mL/min/1.73 m^2^) and 9% had moderate-to-severe CKD (GFR < 60 mL/min/1.73 m^2^). Notably, cyanotic patients are at particular risk: in the same study, 72% of those with Eisenmenger physiology (cyanosis from shunt reversal) had GFR < 90 and 18% had GFR < 60 [2]. Renal dysfunction in ACHD correlates with worse outcomes, independently of cardiac functional class. Dimopoulos et al. reported that moderate/severe CKD was associated with a >3-fold higher mortality hazard in ACHD, even after adjustment. Similarly, others have observed that impaired renal function amplifies the risk of major adverse events in this young population [2].

The main risk factors for CKD in ACHD are advanced age, chronic cyanosis, and the complexity of the heart defect, with single-ventricle and systemic right ventricle lesions being associated with a particularly high risk. A recent analysis found that older age, cyanotic heart disease, and Down syndrome were independent predictors of CKD in ACHD patients [4]. Long-standing hypoxemia leads to hyperviscosity, erythrocytosis, and glomerular injury; multiple cardiac surgeries and cardiopulmonary bypass episodes can cause acute kidney injury (AKI) or nephrotoxic exposures, contributing to later CKD. Moreover, chronic HF itself (with low cardiac output and poor renal perfusion) and venous congestion (elevated central venous pressure) are central drivers of CRS in ACHD. Increased central venous pressure is known to impair renal perfusion and GFR [5], which is particularly relevant in lesions with right HF physiology. ACHD patients may thus exhibit a multisystemic condition in which cardiac and renal dysfunction perpetuate each other [2].

**Figure 1:**
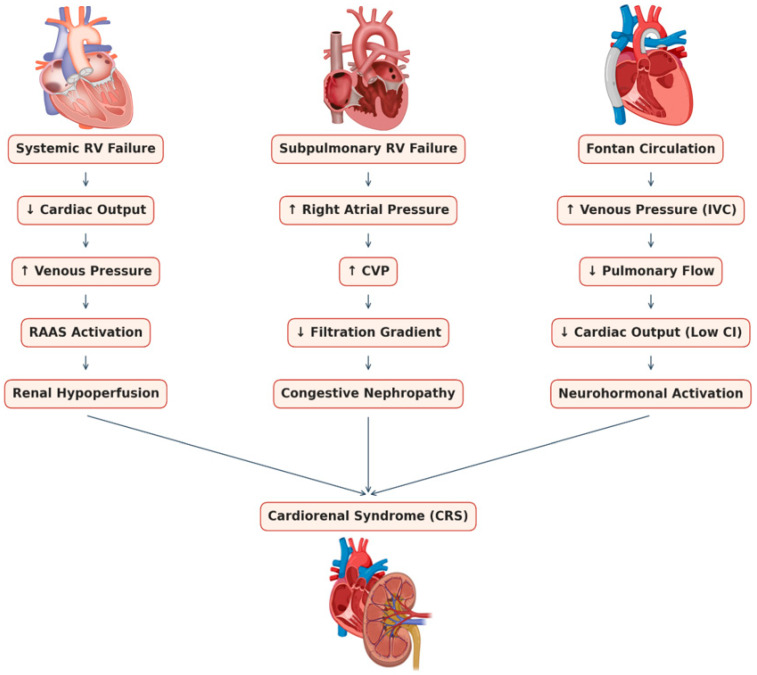
Schematic representation of heart–kidney interactions in various ACHD physiologies, illustrating how systemic RV failure, subpulmonary RV failure, and Fontan circulation each lead to cardiorenal syndrome through distinct mechanisms (low output, venous congestion, hypoxemia, RAAS, or neurohormonal activation).

Despite these concerning figures, awareness of CRS in ACHD is only beginning to grow, while standardized guidelines for monitoring and managing renal function in this population are lacking [3]. Figure 1 illustrates the proposed mechanisms linking congenital cardiac lesions to renal dysfunction in ACHD, and Table 1 summarizes reported prevalence and risk factors of CKD across different ACHD subgroups. In the following sections, we explore CRS in three common physiological contexts: (1) patients with a systemic right ventricle (morphologic RV supporting the systemic circulation), (2) those with a subpulmonary right ventricle who develop right-sided HF, and (3) those with Fontan physiology (single-ventricle circulation). For each, we discuss pathophysiology, clinical presentation, biomarkers, and management strategies, highlighting relevant studies. Understanding these distinctions is vital for the tailored care of ACHD patients with cardiorenal syndrome (Figure 1, Table 1 and Table 2) [1].

In any patient with ACHD, it is essential to identify and manage acquired heart disease risk factors, as these may compound pre-existing risks of heart failure and cardiorenal syndrome (CRS), further complicating clinical outcomes [6].

Table 1 shows the key clinical characteristics of cardiorenal syndrome in ACHD subgroups (systemic RV, subpulmonary RV, Fontan), including the prevalence of renal dysfunction, major risk factors, and recommended management approaches. Each subgroup demonstrates unique challenges, yet all benefit from vigilant renal monitoring and a tailored HF therapy strategy.

**Table 1:** Clinical characteristics of cardiorenal syndrome in ACHD subgroups with references.

CHD Physiology	Prevalence of Renal Dysfunction	Key Pathophysiology	Management Strategies
Systemic RV (e.g., d-TGA with atrial switch, ccTGA)	Moderate to severe in ~30%; albuminuria common [7]	Low systemic output; RAAS activation; RV failure [8,9,10]	Neurohormonal blockade, diuretics, CRT, transplant eval [11,12,13]
Subpulmonary RV (e.g., TOF, Ebstein, Eisenmenger)	Up to 50% with reduced eGFR; albuminuria in edema/TR [2,14]	High CVP; venous congestion; ↓ filtration gradient [5,15]	Volume control, lesion repair, PAH therapy, transplant [2,15]
Fontan circulation	~20–30% with GFR < 90; ~10% with CKD stage 3+ [16]	Chronic venous hypertension; ↓ CO; systemic inflammation [16,17,18]	Cautious diuresis, PLE therapy, ACEi/ARB, transplant eval [19,20]

**Table 2:** Biomarkers and their utility in different ACHD subgroups.

CHD Physiology	Biomarker	Diagnostic Utility	Key References
Systemic RV (e.g., d-TGA with atrial switch, ccTGA)	NT-proBNP	Marker of systemic RV dysfunction and HF severity; correlates with GFR decline	[8,21]
	Cystatin C	More accurate GFR estimation in patients with low muscle mass	[21]
	Albuminuria	Early sign of renal impairment and CRS risk	[7]
Subpulmonary RV (e.g., TOF, Ebstein, Eisenmenger)	BNP/NT-proBNP	Correlates with right atrial and RV pressure; marker of congestion	[14]
	Urine NGAL	Detects tubular injury even with normal creatinine	[22]
	Albuminuria	Associated with venous congestion and systemic edema	[14]
Fontan Circulation	Cystatin C	Detects early GFR decline missed by creatinine-based estimates	[18,21]
	Albuminuria	Reflects chronic renal congestion and low-grade glomerular injury	[18,23]
	BUN:Cr ratio	Suggests prerenal azotemia due to low output state	[23]
	Renin, Aldosterone, Aldosternoe to renis ratio	Reflect neurohormonal activation and guide volume strategies	[24,25]

Abbreviations: ACHD: Adult congenital heart disease, BNP: Brain natriuretic peptide, ccTGA: Congenitally corrected transposition of the great arteries, CHD: Congenital heart disease, CO: Cardiac output, CRT: Cardiac resynchronization therapy, CRS: Cardio renal syndrome, CVP: Central venous pressure, d-TGA: d-Transposition of the great arteries, NGAL: N-Acetyl-β-D-glucosaminidase, PAH: Pulmonary arterial hypertension, PLE: Protein losing enteropathy, RAAS: Renin–angiotensin–aldosterone system, RV: Right ventricle, TOF: Tetralogy of Fallot.

## 2. Systemic Right Ventricle and Cardiorenal Syndrome

Background: A subset of ACHD patients have a morphologic right ventricle (RV) serving as the systemic pumping chamber [26]. This situation occurs in conditions such as d-transposition of the great arteries (d-TGA) repaired with an atrial baffle (Mustard/Senning procedure), or in congenitally corrected transposition of the great arteries (ccTGA) where ventriculoarterial discordance results in the aorta arising from the right ventricle. The systemic right ventricle is inherently poorly equipped to handle long-term systemic afterload, and over time these patients commonly develop systemic RV failure. In adult Mustard/Senning patients, for example, systemic RV systolic dysfunction and HF symptoms become prevalent by the third or fourth decade [27]. Systemic tricuspid (atrioventricular) valve regurgitation, chronically elevated wall stress, and arrhythmias (such as atrial flutter or sinus node dysfunction from atrial scarring) further compound RV failure. This chronic HF state sets the stage for CRS.

Pathophysiology: The failing systemic RV mimics many aspects of left ventricular failure. As the RV’s ejection fraction declines, forward cardiac output to the kidneys drops, activating the renin–angiotensin–aldosterone system (RAAS) and sympathetic nervous system [8]. Neurohormonal activation leads to sodium and water retention, resulting in worsening volume overload. Simultaneously, elevated systemic venous pressure from RV failure can cause hepatic and renal venous congestion [9,10]. However, the hemodynamics can be complex. In atrial-switch TGA, a failing systemic right ventricle causes elevated pulmonary venous pressures, as pulmonary venous return is directed to the systemic RV through the atrial baffle. This leads to pulmonary edema and congestive symptoms similar to left heart failure. In ccTGA without prior repair, systemic RV failure tends to cause systemic venous congestion (ascites, peripheral edema) due to elevated left atrial pressure, which is connected to the failing morphologic RV, while the pulmonary circulation is protected by the morphologic LV. In both scenarios, renal perfusion pressure is compromised by low forward output and possibly by hypotension, while congestion raises renal interstitial pressure—a combination that reduces GFR. Over time, chronic hypoperfusion triggers glomerulosclerosis and impairs the kidney’s adaptive capacity.

Clinical and Biomarker Findings: Patients with systemic RV failure often present with fatigue, exercise intolerance, and signs of HF (edema, liver congestion). Many also have arrhythmias or pacemakers, which can further affect cardiac output and renal perfusion. Laboratory evaluation may reveal rising serum creatinine or blood urea nitrogen, though serum creatinine can underestimate renal dysfunction in ACHD due to lower muscle mass in some patients [21]. Biomarkers like NT-proBNP tend to be elevated in systemic RV HF and correlate with both HF severity and GFR. Some studies suggest cystatin C is a useful adjunct to estimate GFR in ACHD with abnormal body composition [21]. Additionally, albuminuria has been observed as an early sign of renal involvement in these patients. In one cohort of adults with systemic RVs, microalbuminuria was common and associated with a worse New York Heart Association (NYHA) class. This aligns with broader ACHD data showing albuminuria prevalence is highest in those with Fontan circulation or cyanosis but also elevated in systemic RV patients relative to healthy controls [7]. Detecting albuminuria or a declining estimated GFR in a systemic RV patient should prompt intensified monitoring and therapy, as these are harbingers of CRS.

Management Strategies: Managing CRS in systemic RV patients centers on treating HF and protecting the kidneys. Evidence-based HF therapies from acquired heart failure are often applied, although robust ACHD-specific trial data are scarce. Neurohormonal blockade with angiotensin-converting enzyme (ACE) inhibitors or angiotensin receptor blockers (ARBs) is commonly used to reduce afterload on the systemic RV and mitigate RAAS activation [11]. Sodium glucose cotransporter 2 (SGLT2) inhibitors work by reducing cardiac workload, improving energy metabolism, and potentially altering inflammatory pathways [28]. These agents have been demonstrated to be beneficial in the patients with systemic RV dysfunction [12]. Beta-blockers have been studied in small trials (for example, in systemic RV patients with systemic ventricular dysfunction) and led to some improvements in exercise capacity, though no mortality benefit has been proven. Mineralocorticoid receptor antagonists, such as spironolactone, may be prescribed to counteract aldosterone and support diuresis. However, careful monitoring of electrolytes is essential, as many ACHD patients are prone to arrhythmias and electrolyte imbalances can worsen this risk. Diuretics are employed to relieve congestion in the presence of edema or ascites; however, judicious dosing is needed to avoid excessive preload reduction, as systemic RV output may be preload dependent. In these patients, it is crucial to maintain a delicate balance, i.e., sufficient diuresis to relieve the right ventricle and reduce renal venous congestion without causing a significant drop in renal perfusion. Regular monitoring of renal function is advised during the titration of HF medications.

Arrhythmia management is also integral to prevent HF exacerbation and renal dysfunction. Treating atrial tachyarrhythmias with beta-blockers, antiarrhythmics, or catheter ablation can improve hemodynamics and thus renal perfusion (uncontrolled tachycardia can worsen HF and renal function). Bradyarrhythmias, common after Mustard/Senning repairs (due to sinus node dysfunction), may require pacemaker placement or upgrade to cardiac resynchronization therapy (CRT) if dyssynchrony is present. There is evidence that CRT in systemic RV patients can modestly improve systemic RV ejection fraction and clinical status [13], which may in turn improve kidney function by enhancing cardiac output.

Prognosis and Advanced Therapies: If systemic RV failure progresses to end-stage despite medical therapy, evaluation of transplant is recommended. Both heart transplant and heart–lung transplant have been performed in this group. The presence of significant renal dysfunction complicates transplant candidacy. A creatinine clearance below certain thresholds may prompt combined heart–kidney transplant consideration, though data in ACHD are limited. Optimizing renal function through CRS management is therefore essential for keeping transplant options viable. In select cases, mechanical circulatory support (ventricular assist devices) has been used as a bridge to transplant in systemic RV failure, but anatomical challenges (ventricle shape and multiple prior surgeries) make this technically demanding. Thus, timely medical management of CRS and referral for advanced therapies are the keys to improving outcomes in systemic RV patients.

## 3. Subpulmonary Right Ventricle and Renal Implications

Background: The subpulmonary RV refers to the morphological right ventricle supporting the pulmonary circulation (as in a normal heart). Many ACHD patients have a subpulmonary RV that faces abnormal loading conditions due to congenital lesions. Classic examples include repaired tetralogy of Fallot (TOF), where the RV has endured pressure overload before repair and often faces chronic volume overload (pulmonary regurgitation) after repair and Ebstein anomaly of the tricuspid valve—where severe tricuspid regurgitation leads to chronic volume overload of the RV. Additionally, patients with longstanding left-to-right shunts (ASD, VSD) or pulmonary hypertension from any cause impose pressure stress on the subpulmonary RV. Over time, these conditions can lead to right-sided heart failure, even though the left ventricle (systemic ventricle) might be intact. Subpulmonary RV failure is characterized by elevated right atrial pressure and systemic venous congestion with relatively preserved left ventricular output until late stages. This scenario is highly relevant to CRS, as right HF is known to adversely affect renal function via venous congestion. Additionally, RV-LV interactions may result in LV dysfunction purely from RV hemodynamic impairment [29].

Pathophysiology: In isolated RV failure, the principal mechanisms of renal dysfunction are related to elevated central venous pressure (CVP) and reduced effective renal perfusion. A high CVP is directly transmitted to the renal veins and kidney parenchyma, raising interstitial pressure and decreasing the net filtration pressure across the glomerulus [5]. Essentially, when the back-pressure to the kidney is high, it becomes harder for the heart to perfuse the kidneys adequately. This congestive nephropathy is well documented in HF: even moderately elevated jugular venous pressure correlates with worsened GFR [5]. In ACHD, conditions like severe tricuspid regurgitation (as in Ebstein anomaly or pacemaker-induced TR) and chronic pulmonary hypertension (as in Eisenmenger syndrome or primary pulmonary vascular disease) cause persistently high right atrial pressures, which in turn impede kidney filtration. At the same time, if right HF leads to reduced RV output into the pulmonary circulation, left ventricular preload falls, lowering systemic cardiac output and thus renal arterial perfusion. The kidneys, sensing low flow, activate RAAS, causing fluid retention that paradoxically worsens edema and third-spacing. Chronic hypoxemia, seen in cyanotic patients or those with Eisenmenger physiology, introduces an additional complication: secondary erythrocytosis and increased blood viscosity can lead to glomerular microthrombi and progressive fibrosis over time [15]. Uric acid may be elevated (due to both reduced renal clearance and enhanced production in hypoxemia), potentially contributing to gout and further renal insult. Thus, the subpulmonary RV failure creates a milieu of high venous pressure, low forward flow, and neurohormonal excess, a perfect storm for CRS.

Clinical and Biomarkers: Patients with chronic right HF typically present with systemic venous congestion: leg edema, abdominal distension (from ascites), hepatomegaly, and sometimes protein-losing enteropathy (especially in severe tricuspid regurgitation or Fontan, discussed later). Many will have functional renal impairment manifesting as a modest rise in creatinine or reduced eGFR. Notably, because these patients may have normal or even low systemic blood pressure (due to preserved LV function but low output), renal hypoperfusion can be masked until significant. Biomarker-wise, aside from standard renal panels, BNP/NT-proBNP is often elevated in chronic right HF and generally correlates with right atrial pressure and RV end-diastolic pressure; extremely high levels may signal severe hemodynamic compromise affecting the kidneys. Hepatic enzymes (e.g., gamma-GT, bilirubin) are frequently elevated due to congestive hepatopathy and can indirectly indicate the severity of venous congestion impacting the abdominal organs, including the kidneys. Some investigators have measured urinary biomarkers of kidney injury (like N-acetyl-β-D-glucosaminidase or NGAL) in CHD patients with cyanosis, finding evidence of tubular injury even when serum creatinine is normal [22]. Furthermore, microalbuminuria has been observed in right HF; in one study, patients with significant TR and edema had higher urinary albumin excretion than those without congestion, suggesting albuminuria as a marker of systemic congestion [14]. Clinicians should monitor ACHD patients with chronic right HF for rising creatinine and new-onset albuminuria as markers for CRS.

Management Strategies: The cornerstone of managing CRS in subpulmonary RV failure is relieving congestion and optimizing RV function. Diuretic therapy is usually the first step: loop diuretics (e.g., furosemide) to reduce volume overload and venous pressures, promptly improving symptoms and renal blood flow in many cases. Combining with an ACE inhibitor or ARB can be beneficial if blood pressure is adequately maintained, as these agents block RAAS and may protect the kidneys from hyperfiltration injury; however, in isolated right HF, ACEi/ARBs have not been shown to dramatically change RV function and must be used cautiously if systemic hypotension or renal perfusion is borderline. SGLT2 inhibitors, particularly with associated LV dysfunction will be beneficial [12]. Aldosterone antagonists are useful adjuncts, especially if there is evidence of secondary hyperaldosteronism (common in chronic HF). Limiting salt and fluid intake can help control edema and reduce the risk of worsening congestion. However, patients with cyanosis or polycythemia should avoid significant dehydration, as it may increase blood viscosity or lead to shunt thrombosis. Addressing the underlying lesion is critical whenever possible. For example, in repaired TOF with severe pulmonary regurgitation contributing to RV failure, timely pulmonary valve replacement can markedly improve RV size and function, thereby improving hemodynamics and renal perfusion. In Ebstein’s anomaly with massive TR and declining RV function, surgical intervention (tricuspid valve repair or replacement or even cone repair) can relieve volume overload on the RV and reduce CVP, often resulting in improved renal parameters post-operatively. Patients with pulmonary arterial hypertension (PAH) causing RV failure (such as some Eisenmenger physiology patients or those with residual high pulmonary pressures post-repair) might benefit from targeted PAH therapies, e.g., endothelin receptor antagonists, phosphodiesterase-5 inhibitors (sildenafil), or prostacyclin analogs. These can lower pulmonary resistance and RV afterload, which may improve cardiac output and thus kidney perfusion. Indeed, even Eisenmenger patients (previously thought inoperable) on modern PAH therapy have shown improved exercise capacity and end-organ function, though careful monitoring is required [2]. Notably, in true Eisenmenger syndrome, phlebotomy is sometimes performed to manage hyperviscosity; while this can relieve symptoms, overzealous phlebotomy can reduce hemoglobin and oxygen delivery to the kidney, so it must be balanced.

Monitoring and Advanced Care: Regular monitoring of renal function (eGFR, electrolytes) and volume status is needed in chronic right HF patients. This includes monitoring for AKI during intercurrent illnesses or when uptitrating diuretics/ACEi. Collaboration with nephrologists is valuable, for instance, to manage diuretic-resistant edema (adding a thiazide or ultrafiltration in extreme cases) or to prepare for renal replacement therapy if needed. Ultrafiltration can remove fluid in diuretic-refractory HF and may improve diuretic responsiveness by breaking the cycle of congestion. However, in ACHD, decisions about ultrafiltration or dialysis must account for unique anatomy (e.g., shunt physiology or Fontan conduits).

When right HF progresses despite maximal medical and interventional therapy, transplantation is considered. For patients with isolated RV failure and normal pulmonary vascular resistance (e.g., a failed RV in TOF after multiple surgeries), heart transplant is an option that can be lifesaving, often with improvement in renal function post-transplant as perfusion normalizes. In Eisenmenger physiology with severe pulmonary vascular disease, heart–lung transplant may be required; these are complex cases with high perioperative risk, and concomitant renal dysfunction must be carefully managed before and after the transplant. The presence of significant CKD (e.g., GFR <40 mL/min) in any ACHD patient raises the question of combined organ transplantation, but combined heart–kidney or heart–lung–kidney transplants are exceedingly rare and considered only on a case-by-case basis. Thus, timely cardiac intervention, along with control of contributing risk factors like hypertension and diabetes when present, is essential to halt the progression of CRS.

## 4. Fontan Physiology and the Kidney

Background: The Fontan operation, used for single-ventricle congenital heart defects, results in a unique physiology where the systemic venous return is routed directly to the pulmonary arteries without a subpulmonary ventricle. The Fontan circulation creates a total cavopulmonary connection that is effective in childhood, but it is inherently non-physiological. By adulthood, many Fontan patients develop “Fontan syndrome”, a constellation of issues including Fontan-associated liver disease (FALD), coagulopathy, protein-losing enteropathy (PLE), and Fontan-associated nephropathy. Key hemodynamic features in Fontan patients are elevated central venous pressure and chronic low cardiac output. These features have direct consequences for renal function. Indeed, the kidneys of a Fontan patient have been working against a chronically elevated venous pressure since the Fontan was established (often in early childhood) and often at a suboptimal perfusion pressure. Over time, this leads to structural and functional renal changes. Studies in adolescent and adult Fontan patients have begun to quantify this: by late adolescence/young adulthood, approximately 20% of Fontan patients have an eGFR below 90 (mL/min/1.73 m^2) [16] and about 10% demonstrate moderate CKD (eGFR < 60) in some series [16]. While mild renal dysfunction in Fontan patients may be “well tolerated” in the short term [16], concern grows as these patients age into their 30s and 40s, since the decline in organ function may accelerate.

Pathophysiology: The high systemic venous pressure in Fontan circulation (often 15–20 mmHg in the IVC and hepatic veins) is transmitted to renal veins, raising the pressure within the glomerular outflow tract. This chronic venous hypertension leads to congestion and edema in the kidney’s interstitium, impairing glomerular filtration (similar to the mechanism in isolated right HF but even more pronounced given the lack of a pumping ventricle for the pulmonary circuit). Additionally, cardiac output is limited in Fontan patients; without a subpulmonary ventricle, pulmonary blood flow (and hence preload to the systemic ventricle) depends on the pressure gradient and transpulmonary flow, which is inherently restricted. Many Fontan patients have a chronically low cardiac index [17,30]. This diminished renal perfusion pressure chronically stimulates RAAS, contributing to fluid retention, vasoconstriction, and renal fibrosis. Another factor is the chronic state of low-grade hypoxemia and systemic inflammation—Fontan patients often have AV malformations and mildly reduced arterial oxygen saturation, which can cause renal cortical hypoxia over time [18,31,32,33]. Furthermore, complications like PLE (protein-losing enteropathy) can lead to hypoalbuminemia, reducing plasma oncotic pressure and exacerbating edema in tissues including the kidney. Low albumin levels can also directly impact glomerular pressure dynamics and promote nephrotic-range proteinuria. Liver congestion in Fontan patients may result in cirrhosis and impaired synthesis of proteins important for renal perfusion (like angiotensinogen and albumin), although the interplay is complex. In summary, Fontan physiology imposes a triple insult on kidneys: venous congestion, hypoperfusion, and secondary neurohormonal/inflammatory activation.

Clinical and Biomarker Profile: Several biomarkers reflect renin–angiotensin–aldosterone system (RAAS) activation, including elevated plasma renin activity (PRA) and total renin levels, which have been linked to a poor diuretic response in heart failure patients [24]. Angiotensin II, a key RAAS effector, promotes vasoconstriction and stimulates aldosterone secretion, which furthers sodium retention and fibrosis [25]. Elevated aldosterone levels and the aldosterone-to-renin ratio (ARR) are commonly used to assess RAAS status and guide therapy. These biomarkers are valuable in identifying maladaptive RAAS activation and tailoring interventions to slow cardiorenal deterioration.

Clinicians managing adult Fontan patients should have a high index of suspicion for renal dysfunction, even if serum creatinine appears “normal” for age. Many Fontan patients are relatively young and have low muscle mass, so creatinine can underestimate the degree of GFR reduction. Measurements of cystatin C have been shown to detect lower GFR in Fontan patients that creatinine-based formulas might miss [21]. Albuminuria is a common finding: one study found that approximately 10–25% of Fontan patients have microalbuminuria or proteinuria by their early adulthood [18]. Importantly, even clinically “well” Fontan patients (NYHA I–II) may have significant albuminuria and reduced kidney functional reserve [23]. This suggests renal injury begins early in the Fontan timeline. Other markers, such as elevated BUN or a high BUN/Cr ratio, may indicate prerenal azotemia from low output. Fontan patients often have mildly elevated liver enzymes and bilirubin from congestion; if the liver is significantly cirrhotic, hepatorenal physiology (similar to cirrhosis-induced renal dysfunction) can compound CRS. Figure 1 illustrates the multi-organ interactions in Fontan circulation failure, including the kidney. On imaging, the kidneys in Fontan patients might appear slightly enlarged and congested on ultrasound, and Doppler studies can show pulsatile, elevated venous pressures in renal veins. Biopsy data are limited, but small series have reported congestion, fibrosis, and glomerular basement membrane changes in Fontan kidneys [34]. From a symptom standpoint, Fontan patients with significant CRS may have refractory edema (often anasarca if PLE is present), chronic fatigue, and worsened exercise tolerance. Some may progress to needing supplemental oxygen due to pulmonary arteriovenous malformations (which indirectly can worsen systemic oxygen delivery to organs, including the kidneys).

Management Strategies: Managing CRS in Fontan patients is challenging and requires a nuanced approach. The usual HF toolbox must be applied carefully.

- Volume management: Diuretics are frequently used to manage edema and effusions in Fontan patients. Loop diuretics (often in combination with aldosterone antagonists) can reduce venous congestion. However, over-diuresis can be detrimental: Fontan circulation is highly preload dependent and excessive volume removal may precipitate a low cardiac output state and acute kidney injury. Therefore, clinicians often aim for a “euvolemic” state rather than aggressive diuresis. Monitoring daily weights, renal function, and even invasive hemodynamics (during catheterization) guides therapy. Some Fontan patients benefit from outpatient intravenous albumin infusions (in those with PLE) combined with diuretics to augment plasma volume and diuresis effectiveness.
- Afterload reduction: If the systemic ventricle in a Fontan (usually a single ventricle that could be morphologically left or right) has systolic dysfunction or systemic hypertension, ACE inhibitors or ARBs are used to reduce afterload. Evidence for ACE-inhibitor benefit in Fontan patients without ventricular dysfunction is limited—small trials did not show improved exercise capacity in pediatric Fontan patients on enalapril, for example [35]. But in adults, if blood pressure is elevated or there are signs of proteinuric kidney disease, an ACEi/ARB is often added for its renal-protective effects (analogous to use in other CKD) [36]. Cautious titration is needed to avoid hypotension.
- Evolving role of SGLT2 inhibitors: SGLT2 inhibitors have been demonstrated to reduce the incidence of cardiovascular death and heart failure in patients with reduced ejection fraction (HFrEF) and preserved ejection fraction (HFpEF) [37]. SGTL2 inhibitors lower blood glucose and glycated hemoglobin in patients with type 2 diabetes, improve cardiac function in patients with heart failure with or without type 2 diabetes, and improve renal function [19]. These agents have been demonstrated to be safe and beneficial in Fontan circulation [38,39]. Metabolic syndrome is common among patients with Fontan circulation and may also be associated with poor hemodynamics [40]. SGTL2 inhibitors may offer additional benefits in these patient phenotypes of Fontan physiology and heart failure [20]. Initiation of dapagliflozin is indicated in patients with an eGFR > 25 mL/min/1.73 m2 [41]. While the addition of SGLT2 inhibitors to standard diuretic therapy increases urine output without impacting renal function, it is also critical to exercise caution and closely monitor renal function [42]. SGTLT2 agents significantly reduce the diuretic requirement, and it may be prudent to minimize the diuretic use when using these agents [43].
- Pulmonary vasodilators: Given the absence of a subpulmonary pump, reducing pulmonary vascular resistance can significantly impact Fontan flow. Medications like sildenafil or bosentan (an endothelin blocker) have been tried in Fontan patients to improve exercise capacity and cardiac output [44,45,46]. Some studies have shown modest improvements in hemodynamics, though others are inconclusive. Still, if pulmonary vascular resistance is elevated or there is suspicion of impaired pulmonary blood flow, a trial of pulmonary vasodilator therapy may be considered. Improved pulmonary flow should enhance preload to the systemic ventricle and potentially renal perfusion. These therapies can also improve exercise tolerance, which indirectly benefits overall health, including kidney perfusion during activity.
- Mineralocorticoid antagonists: In patients with chronic systolic heart failure and mild symptoms (NYHA class II), eplerenone significantly reduced the risk of cardiovascular death or heart failure hospitalization compared to placebo [47]. The use of eplerenone in patients with Fontan circulation experiencing heart failure has not been extensively studied. Given the limited and inconclusive data, the routine use of eplerenone in Fontan patients with heart failure cannot be recommended at this time. Further research is necessary to establish its safety and efficacy in this specific group.
- Management of complications: Treating Fontan-specific complications can mitigate CRS. For instance, if a patient has significant Fontan obstruction (e.g., a narrowed conduit or collaterals causing shunting), relieving this via catheter intervention or surgery can improve hemodynamics. In patients with PLE, therapies such as steroids, heparin, or novel lymphatic interventions (like thoracic duct decompression) may reduce protein loss, raising albumin levels and helping restore intravascular volume, thus improving renal blood flow. Liver congestion and cirrhosis are harder to directly treat; however, avoiding hepatotoxins and monitoring for HCC (hepatocellular carcinoma) is important, as advanced liver disease can further impair kidney function (via hepatorenal mechanisms).
- Surveillance: Fontan patients require lifelong surveillance, and renal function should be part of routine follow-up. We recommend at least annual serum creatinine/eGFR checks and periodic urinalysis for albumin. When GFR falls below ~60, consultation with a nephrologist is prudent to manage CKD risks (bone health, anemia, electrolyte disturbances) and to discuss renoprotective measures. Aggressive control of blood pressure (often Fontan patients have low BP, but if normal or high, keep it in optimal range) and avoidance of nephrotoxic drugs (NSAIDs, certain antibiotics) are general principles.

Advanced Therapies: As Fontan patients reach adulthood, some will develop “Fontan failure”—A state of circulatory collapse characterized by protein-losing enteropathy, refractory ascites, plastic bronchitis, and cachexia. In this setting, multi-organ transplantation is often considered. The standard approach for a failing Fontan in an adult is heart transplantation with or without adjunct organ transplants. Isolated heart transplant can be performed, expecting that liver and kidney function will improve once normal two-ventricle physiology is restored. However, long-standing Fontan may cause irreversible liver cirrhosis and renal damage. Some centers therefore opt for combined heart–liver transplant in Fontan patients with advanced hepatic fibrosis. Kidney transplant along with the heart (or heart–liver) is less common but has been reported in cases of severe CKD. The threshold for adding a kidney transplant is if renal dysfunction is unlikely to recover (for example, a pre-transplant GFR well below 40 and significant renal fibrosis on biopsy). Because organ availability is limited, careful patient selection is essential. Mechanical support as a bridge in Fontan patients is very complex due to the lack of a subpulmonary ventricle and abnormal flow dynamics—in rare cases an axial-flow pump has been placed in the IVC to aid Fontan flow, but this remains experimental. Thus, early referral for transplant evaluation, before end-organ damage is irreversible, is a key strategy. Encouragingly, some data suggest that mild renal dysfunction in Fontan patients can remain stable for several years [16], meaning there is a window of opportunity for intervention.

## 5. Conclusions

Adults with congenital heart disease (ACHD) are increasingly affected by cardiorenal syndrome (CRS), which significantly influences prognosis across all physiological subgroups. Whether due to systemic right ventricular overload, subpulmonary right heart failure with venous congestion, or the unique Fontan circulation, renal impairment is common and strongly associated with worse outcomes. Managing CRS in ACHD requires a multidisciplinary, individualized approach that goes beyond traditional heart failure care. Clinicians must balance hemodynamics, neurohormonal blockade, and volume status with renal protection strategies. Despite the growing burden, formal guidelines for renal monitoring in ACHD remain lacking. Future research should prioritize biomarker validation, optimal screening protocols, and the incorporation of renal outcomes in ACHD heart failure trials. Closing these knowledge gaps is essential to improving long-term survival and quality of life in this complex population.

CRS in adult congenital heart disease epitomizes the need for holistic, organ-crossing care. By maintaining vigilance for renal issues, implementing timely management, and pursuing collaborative care models, we can better address this pernicious coupling of heart and kidney failure. With appropriate strategies and further research, there is hope to mitigate CRS and thereby improve the longevity and well-being of ACHD patients as they continue to break new ground in survivorship.

## Data Availability

Not applicable.

## Glossary

ACHD	Adult Congenital Heart Disease
CRS	Cardiorenal Syndrome
CKD	Chronic Kidney Disease
GFR	Glomerular Filtration Rate
eGFR	Estimated Glomerular Filtration Rate
NT-proBNP	N-terminal pro-B-type Natriuretic Peptide; a biomarker for heart failure
BNP	B-type Natriuretic Peptide
NGAL	Neutrophil Gelatinase-Associated Lipocalin; a marker of acute kidney injury
RAAS	Renin–Angiotensin–Aldosterone System
ARR	Aldosterone-to-Renin Ratio
CRT	Cardiac Resynchronization Therapy
PLE	Protein-Losing Enteropathy
TOF	Tetralogy of Fallot
TGA	Transposition of the Great Arteries
ccTGA	Congenitally Corrected Transposition of the Great Arteries
d-TGA	Dextro-Transposition of the Great Arteries
PAH	Pulmonary Arterial Hypertension
TR	Tricuspid Regurgitation
CO	Cardiac Output
CVP	Central Venous Pressure
ACEi	Angiotensin-Converting Enzyme Inhibitor
ARB	Angiotensin II Receptor Blocker
HF	Heart Failure
